# Arterial stiffness in hypertensive and type 2 diabetes patients in Ghana: comparison of the cardio-ankle vascular index and central aortic techniques

**DOI:** 10.1186/s12902-016-0135-5

**Published:** 2016-09-29

**Authors:** Kwame Yeboah, Daniel A. Antwi, Ben Gyan, Virginia Govoni, Charlotte E. Mills, J. Kennedy Cruickshank, Albert G. B. Amoah

**Affiliations:** 1 Department of Physiology, School of Biomedical & Allied Health Sciences, University of Ghana, P.O. Box KB 143, Accra, Ghana; 2 Department of Immunology, Noguchi Memorial Institute for Medical Research, University of Ghana, Accra, Ghana; 3 Cardiovascular Medicine Group, Division of Diabetes and Nutrition, King’s College and King’s Health Partners, London, UK; 4 Department of Medicine and Therapeutics, School of Medicine and Dentistry, University of Ghana, Accra, Ghana; 5 National Diabetes Management and Research Centre, Korle-Bu Teaching Hospital, Korle-Bu, Accra, Ghana

**Keywords:** Cardio-ankle vascular index, Arterial stiffness, Aortic pulse wave velocity, Diabetes, Hypertension, Ghana

## Abstract

**Background:**

Diabetes and hypertension increase arterial stiffness and cardiovascular events in all societies studied so far; sub-Saharan African studies are sparse. We investigated factors affecting arterial function in Ghanaians with diabetes, hypertension, both or neither.

**Method:**

Testing the hypothesis that arterial stiffness would progressively increase from controls to multiply affected patients, 270 participants were stratified into those with diabetes or hypertension only, with both, or without either. Cardio-ankle vascular index (CAVI), heart–ankle pulse wave velocity (haPWV), aortic PWV (PWVao) by Arteriograph, aortic and brachial blood pressures (BP), were measured.

**Results:**

In patients with both diabetes and hypertension compared with either alone, values were higher of CAVI (mean ± SD, 8.3 ± 1.2 vs 7.5 ± 1.1 and 7.4 ± 1.1 units; *p* < 0.05), PWVao (9.1 ± 1.4 vs 8.7 ± 1.9 and 8.1 ± 0.9 m/s; *p* < 0.05) and haPWV (8.5 ± 1 vs 7.9 ± 1 and 7.2 ± 0.7 m/s; *p* < 0.05) respectively. In multivariate analysis, age, having diabetes or hypertension and BMI were independently associated with CAVI in all participants (β = 0.49, 0.2, 0.17 and -0.2 units; *p* < 0.01, respectively). Independent determinants of PWVao were heart rate, systolic BP and age (β = 0.42, 0.27 and 0.22; *p* < 0.01), and for haPWV were systolic BP, age, BMI, diabetes and hypertension status (β = 0.46, 0.32, -0.2, 0.2 and 0.11; *p* < 0.01).

**Conclusion:**

In this sub-Saharan setting with lesser atherosclerosis than the western world, arterial stiffness is significantly greater in patients with coexistent diabetes and hypertension but did not differ between those with either diabetes or hypertension only. Simple, reproducibly measured PWV/CAVI may offer effective and efficient targets for intervention.

**Electronic supplementary material:**

The online version of this article (doi:10.1186/s12902-016-0135-5) contains supplementary material, which is available to authorized users.

## Background

Epidemiological transition has been underway for many years in most lower/middle income countries, as in Ghana. Chronic adult diseases including hypertension, obesity, resulting metabolic syndrome and diabetes are now common [1, 2]. Consequently, premature macro- and micro-vascular conditions, including hypertensive heart failure (if not yet much coronary disease), stroke, renal failure, and arterial complications of Type 2 diabetes (T2D) now burden patients and health services [3, 4]. Arterial stiffness is a powerful predictor of such future cardiovascular (CV) complications and all-cause mortality in all patient groups studied, even after adjusting for age, blood pressure (BP) or other risk factors [5, 6]. In addition, arterial stiffness is one of the earliest detectable manifestations of adverse structural and functional changes within the vessel wall [7]. The gold standard for measuring arterial (aortic) stiffness is carotid-femoral pulse wave velocity [8]. Cardio-ankle vascular index (CAVI), an index of arterial stiffness developed in Japan from measuring heart-ankle PWV (haPWV) and brachial BP, is reported to be independent from BP at the time of measurement; hence CAVI may indicate organic stiffness of the arterial wall, minimising the influence of BP fluctuations [9].

There is little arterial function research in sub-Saharan Africa population, and none to our knowledge, on CAVI and haPWV in patients with T2D or hypertension [10]. We used CAVI and another calibrated cuff-based device, the Arteriograph, to measure aortic PWV to test the hypothesis that arterial stiffness would progressively increase with increasing burden of CV risk, mainly as having hypertension or Type 2 diabetes in Ghanaians.

## Methods

### Study design and patients

The study was performed at the National Diabetes Management and Research Centre, Accra, established in 1995 as a centre of excellence for diabetes research, management and education in Ghana. In all, 300 participants, aged 30–75 years, without type 1 diabetes or overt CVDs, were recruited as every 3^rd^ person attending clinics. Healthy volunteers from the community who were glucose tolerance tested and did not have T2D served as controls. There were 210 T2D patients, with 90 age-matched non-diabetes volunteers. In the final analysis, 30 out of the 300 participants were excluded: 24 due to ankle-brachial index <0.90 or other poor arterial wave tracings; 6 had intermediate fasting plasma glucose (FPG) or 2-h post 75 g glucose load plasma glucose (2 h-PPG) above the threshold for non-diabetes. The remaining sample of 270 included 192 T2D patients, 143 with and 49 without defined ‘hypertension’ (BP ≥140 or 90 mmHg or on treatment); 50 hypertensives without T2D and 28 controls without hypertension or diabetes. T2D status was clinical, based on not requiring insulin at diagnosis of diabetes and older age of presentation (>35 years). Ethical approval came from the University of Ghana Medical School Ethical and Protocol Review Committee (Protocol ID number: MS-Et/M.2 – P.4.10/2012-2013); all participants gave written informed consent after thorough explanation of the procedures involved.

### Anthropometric measurements

Using standard protocols [11], waist and hip circumferences were measured in duplicate with a non-elastic tape, maximum height to the nearest 0.1 cm using a Stadiometer, and weight to the nearest 0.1 kg on a digital, heavy-duty floor scale (Seca, Hamburg, Germany). Percentage body fat was assessed using the Body composition monitor (BF- 508, Omron Healthcare, Inc., Vernon Hills, IL, USA).

### Biochemical analysis

Blood samples were drawn in the morning after 8–12 h of overnight fasting into plain vacuum tubes to measure plasma lipids and fluoride oxalate tubes for glucose levels. FPG, 2-h PPG, total cholesterol (TC), high-density lipoprotein cholesterol (HDL) and triglyceride (TG) levels were analysed by colorimetric enzymatic assays using BS 120 chemical autoanalyser (Mindray, China) and commercial reagents (Randox Laboratory Reagents, UK). Low-density lipoprotein cholesterol levels were calculated using Friedewald’s formula [12]. All analyses were performed at the Diabetes Research and Chronic Disease Reference Laboratory.

### Cardiovascular measurements

Brachial and aortic pressure indices, aortic PWV (PWVao), and aortic augmentation index (AIx) were measured with Arteriograph (TensioMed Kft., Hungary), with the subject lying supine, after 10 min rest in a temperature controlled room (22 ± 2 °C). The Arteriograph cuff was applied on the right arm over the brachial artery to detect arterial wall oscillations in the upper arm using the ‘stop-flow’ principle previously described [13], and analysed using dedicated software.

CAVI and haPWV were measured using the Vasera 1500 N (Fukuda-Denshi, Japan) with the participant resting supine for at least 10 min before the measurement. Electrocardiogram electrodes were placed on both wrists, a microphone for detecting heart sounds on the sternum, and cuffs were wrapped around both upper arms and above each ankle. CAVI values were computed automatically. Briefly, CAVI corresponds to the stiffness parameter β, calculated from values of heart-ankle PWV and BP as follows;

$$ \upbeta =\left(2\uprho /\Delta \mathrm{P}\right)\ \left[\mathrm{In}\ \left({\mathrm{P}}_{\mathrm{s}}/{\mathrm{P}}_{\mathrm{d}}\right)\right]{\mathrm{P}\mathrm{WV}}^2 $$

Where ρ indicates blood density; ΔP, pulse pressure; In, natural log; P_s_, systolic BP; and P_d_, diastolic BP [9, 14].

CAVI and Arteriograph measures were done in random order from a digital algorithm.

### Statistical analysis

IBM SPSS version 20 was used to summarise results as proportions for categorical variables and means and standard deviations (SD) for continuous variables. Mean differences between groups of patients were analysed by ANOVA, categorical data by χ^2^ test, and association between variables using Pearson’s correlation. Multiple regressions, with all appropriate parameters (eg, just 1 BP type, systolic or pulse pressure etc) forced into the model, were performed to determine independent determinants of CAVI, aortic PWV and haPWV. *p*-values < 0.05 were considered statistically significant.

## Results

### Characteristics of study participants

Clinical and haemodynamic characteristics of 270 participants with quality waveforms showed no significant difference in age, gender or former smoking, although controls were somewhat younger (Table 1). As expected, hypertensive T2D patients had the highest PWVao, greater than those with T2D only and hypertension only; both these groups had higher PWVao than controls. Similarly, hypertensive T2D had significantly higher CAVI and haPWV than hypertensives only, T2D only and controls. Despite their marginally higher BPs, the ‘healthy’ controls had lower PWVao, CAVI and haPWV than patients with T2D alone. Aortic pressure indices in hypertensive T2D and hypertensive only patients were also higher than in T2D only patients. T2D patients had lower total and LDL cholesterol (with statin treatment as shown) than non-diabetes patients (Additional file 1: Table S1).

**Table 1:** Clinical characteristics of participants

Characteristics	Controls (*n* = 28)	T2D only (*n* = 49)	Hypertensive only (*n* = 50)	Hypertensive T2D (*n* = 143)
Age	51.5 ± 11.8	52.8 ± 8.7	56.1 ± 9.4	57.1 ± 9.7
Male gender, n (%)	15 (53.6)	33 (67.3)	17 (34)	64 (44.8)
BMI (kg/m^2^)	28.8 ± 5.2	26.1 ± 3.6	30 ± 5.6	29.7 ± 5.3
Ex-smokers, n (%)	5 (17.9)	6 (12.2)	11 (22)	5 (3.4)
Medications n (%)				
on Antihyper-tensive Treatment	NA	NA	39 (78)	90 (62.9)
on insulin		7 (14.3)		45 (31.5)
on oral hypoglycaemics		10 (28.6)		39 (27.3)
On insulin plus oral hypoglycaemics		20 (40.8)		59 (41.3)
On Statins	0	23 (46.9)	6 (12)	38 (26.6)
Duratn.T2D (yrs)	NA	7 ± 6	NA	11 ± 8^#^
Vasera Measures				
SBP (mm Hg)	128 ± 15	125 ± 29	150 ± 29^*#^	149 ± 34^*#^
DBP (mm Hg)	80 ± 7	83 ± 8	93 ± 12^*#^	92 ± 10^*#^
PP (mm Hg)	48 ± 11	47 ± 9	57 ± 14^*#^	63 ± 15^*#^
MBP (mm Hg)	96 ± 9	99 ± 9	112 ± 14^*#^	112 ± 12^*#^
H Rate (bpm)	64 ± 10	74 ± 11	69 ± 11	78 ± 13
CAVI^a^	6.9 ± 1	7.4 ± 1.1^*^	7.5 ± 1.1^*^	8.3 ± 1.2^*§#^
haPWV (m/s) ^a^	6.9 ± 0.8	7.2 ± 0.7^*^	7.9 ± 1^*^	8.5 ± 1^*§#^
Arteriograph Measures				
Brachial Pressure				
SBP	124 ± 10	123 ± 20	144 ± 35*^#^	152 ± 30*^#^
DBP	74 ± 9	75 ± 9	88 ± 13*^#^	88 ± 13*^#^
PP	50 ± 6	51 ± 7	62 ± 12*^#^	67 ± 14*^#^
MBP	90 ± 9	92 ± 8	108 ± 14*^#^	110 ± 14*^#^
HR	65 ± 10	73 ± 16	66 ± 22	75 ± 16
Aortic Pressure				
Aortic SBP	120 ± 12	116 ± 12	148 ± 27*^#^	151 ± 26*^#^
Aortic PP	46 ± 7	39 ± 14	65 ± 17*^#^	58 ± 25*^#^
Aortic AIx	29 ± 13	19 ± 11	38 ± 11*^#^	30 ± 15*^#§^
Aortic PWV	7.8 ± 1.1	8.1 ± 0.9^*^	8.7 ± 1.9*	9.1 ± 1.4^*#§^

*T2D* type 2 diabetes, *BMI* body mass index, *CAVI* cardio-ankle vascular index, *haPWV* heart-ankle pulse wave velocity, *SBP* systolic blood pressure, *DBP* diastolic blood pressure, *PP* pulse pressure, *MBP* mean blood pressure, *HR* heart rate, *PWV* pulse wave velocity, *AIx* augmentation index

^a^Adjusted for age and gender

*vs Controls, *p* < 0.05

§ vs Hypertensive only, *p* < 0.05

# vs T2D only, *p* < 0.05

### Correlation between indices of arterial stiffness and patient parameters

In univariate analysis, CAVI was associated with age, waist-hip ratio, pulse and mean BP in all participants. For combined patients group, CAVI was also associated with body fat. In controls, CAVI was positively associated with age and fasting triglycerides levels. However, in all groups, CAVI was not associated with systolic/diastolic BPs, heart rate and plasma glucose and lipid profile. (Additional file 1: Table S2). In all participants and combined patient groups, PWVao was associated with age, BMI, body fat, systolic/diastolic BPs, mean BP, pulse pressure and heart rate. In controls, PWVao was associated with age only (Additional file 1: Table S3). Heart-ankle PWV was associated with age, waist-hip ratio, systolic/diastolic BPs, pulse pressure and mean BP in both all participants and combined patient groups (Additional file 1: Table S4).

**Fig. 1:**
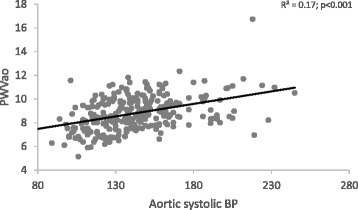
Aortic stiffness across various aortic systolic BP levels

**Fig. 2:**
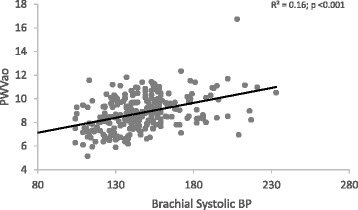
Aortic stiffness across various brachial systolic BP levels

When PWVao was plotted against the range of aortic systolic BPs (Fig. 1), at any level of BP in all groups, there were participants with lower BP who had stiffer arteries, as was also found with brachial pressures (Fig. *2*). Similar observations were also made with a plot of CAVI against brachial systolic BP (Additional file 2: Figure S2), even if there was no significant relationship between them. At lower BP levels in all groups, some individuals had higher levels of ‘intrinsic’ arterial stiffness independent of BP, which is CAVI. (Additional file 2: Figure S1).

### Multiple linear regressions

In multiple regression analyses (Table 2), in all participants and combined patients groups, CAVI was positively and independently related to age, most strongly, then to hypertension &/or diabetes status, WHR and prominently negatively to BMI. However, in controls, only age was a determinant of CAVI. Systolic BP made no significant contribution to variation in CAVI. When systolic BP was substituted with other brachial pressures – diastolic, mean or pulse – none were significantly associated with CAVI (data not shown – but see Additional file 2: Figure S2 for Systolic BP vs CAVI plot).

**Table 2:** Multiple linear regression analysis for CAVI and subject parameters

	Entire group		Control group		Patient groups combined	
	β	p	β	p	β	p
Age	0.49	<0.01	0.49	0.04	0.48	<0.01
Gender	0.06	0.281	-0.19	0.2	0.09	0.131
HTN status	0.2	<0.01			0.2	0.01
BMI	-0.2	<0.01	0.08	0.34	-0.25	<0.01
T2D status	0.17	<0.01			0.13	0.01
WHR	0.09	0.09	0.08	0.74	0.18	<0.01
SBP *	0.09	0.1	0.21	0.23	0.1	0.11
Heart rate	0.04	0.45	-0.23	0.29	0.07	0.23

β = standardised regression coefficient

* = Values for Pulse pressure and mean BP were also not significant

*HTN* hypertension, *T2D* type 2 diabetes, *BMI* body mass index, *CAVI* cardio-ankle vascular index, *SBP* systolic blood pressure, *HR* heart rate

For haPWV (Table 3), in all participants and combined patients group, systolic BP, age and hypertension status had positive relationships and again BMI made a negative contribution. T2D status was independently associated with haPWV in all participants but not in combined patients group. In controls, age and systolic BP contributed significantly to haPWV. Heart rate, systolic BP and age each contributed significantly to variation in PWVao in all participants, but none of the cardiovascular risk factors were independently associated with PWVao in the controls.

**Table 3:** Multiple regression analysis between aortic PWV, haPWV and subject parameters

	Aortic PWV						ha-PWV					
	Entire group		Control group		Patient group		Entire group		Control group		Patient group	
	β	P	β	p	β	p	β	p	β	p	β	p
Heart rate	0.42	<0.001	0.35	0.36	0.27	<0.001	0.05	0.32	-0.06	0.74	0.07	0.18
SBP	0.27	<0.001	0.25	0.26	0.41	<0.001	0.46	<0.01	0.58	<0.01	0.47	<0.01
Age	0.22	<0.001	0.62	0.09	0.22	0.001	0.32	<0.01	0.45	0.04	0.31	<0.01
BMI	0.09	0.142	0.01	0.97	0.09	0.2	-0.2	<0.01	-0.11	0.62	-0.24	<0.01
Gender	0.12	0.05	0.32	0.065	0.1	0.14	0.04	0.402	-0.21	0.11	0.07	0.23
HTN status	-0.03	0.361			-0.03	0.23	0.2	<0.01			0.19	<0.01
T2D status	0.01	0.391			0.01	0.29	0.11	0.02			0.09	0.12
WHR	0.01	0.88	0.02	0.95	-0.04	0.51	0.08	0.09	0.03	0.86	0.16	0.01

*HTN* hypertension, *T2D* type 2 diabetes, *BMI* body mass index, *WHR* waist-hip ratio, *CAVI* cardio-ankle vascular index, *SBP* systolic blood pressure, *HR* heart rate

## Discussion

Our findings suggest that these indices of arterial stiffness, CAVI, haPWV from which CAVI is derived, and PWVao are significantly increased in Ghanaian patients with diabetes and hypertension more than in those with either alone, or without both conditions. Our data here are the first from a sub-Saharan African patient population where generally, atheromatous disease is still uncommon; haemorrhagic stroke, hypertensive heart and renal failure remain the major causes of vascular events [15]. There was no difference in CAVI and PWVao values between diabetes without hypertension, and hypertension patients without diabetes, suggesting that both conditions are similarly related to arterial pathology, even when the CAVI computation adjusts for BP differences. That conclusion is supported by the similar heart-ankle pathway PWV data, which has to be extracted from the software (based on the time delay between the phonocardiogram’s 2^nd^ sound to arrival of the pressure wave at the ankle). While the PWV of the so-called brachial-ankle arterial pathway, in general use in Japan, is prognostic [16], its cardiac-brachial component is not.

The key importance of arterial stiffness is that its prognostic value persists when other known risk factors including any BP (systolic, pulse or mean) have been taken into consideration [17]. Cruickshank et al. [18] showed how arterial stiffness, measured by aortic PWV, predicted mortality in T2D patients, in those with ‘impaired’ glycaemia and in glucose-challenged controls and hence that arterial PWV could be considered an integrated index of vascular health. That sample included African-Caribbeans, who still have lower coronary heart disease, but not stroke, risk than the British population average; similar to but not as extreme a difference as in Ghana. CAVI, a novel index, is thought to reflect collective overall compliance in the aorta, femoral and popliteal arteries, independent of BP, based on the formula quoted above (Methods) [9]. CAVI has also been shown to be superior to ankle-brachial PWV in predicting cardiovascular events. This superiority of CAVI had been attributed to its independence, theoretically, from BP at the time of measurement, so that CAVI may indicate the intrinsic stiffness of the arterial wall [9, 14, 19]. CAVI may also be a useful screening tool for moderate to advanced levels of arterio-, rather than athero-sclerosis [20], the major difference between traditionally high ‘BP’- and plaque- driven pathology. We found that patients with co-existent diabetes and hypertension have dramatic reduction arterial compliance, or increase stiffness, than patients with single condition. The lack of difference in CAVI/haPWV but a clearer difference in the Arteriograph’s PWVao between the T2D *without* hypertension and hypertension group *without* T2D suggest less arteriosclerosis in the more muscular peripheral arteries in the legs here. In indigenous black South Africans, where smoking rate and atherosclerosis remain (currently) low, arterial stiffness as carotid-femoral PWV is still pressure-dependent [21]. Such data illustrate how PWV generally and CAVI may add value to a routine BP ‘check’. Without sub-Saharan intervention trials, their routine use will not yet be judged ‘cost-effective’.

Most studies where arterial stiffness was evaluated using CAVI have been performed in non-African, South-East Asian populations [9, 14, 19, 20]. In agreement with our findings, Wang et al [22] showed that CAVI is elevated in a Chinese population with coexistent hypertension and diabetes. The dynamics of CAVI, as an index of arterial stiffness in sub-Saharan Africans, may differ from that in Asians or Caucasians [23], as found by Uurtuya *et al* in comparable healthy young subjects [24] and hypertensive T2D patients in Japan and Mongolia [25]. Arterial stiffness measured by PWV here should reflect arterial function in these patient samples, but due to the relatively small number of controls, estimating general stiffness in sub-Saharan African populations at large will need community-based population studies.

The major determinants of PWVao in this study were heart rate, systolic BP and age. PWV, as a measure of arterial stiffness, is affected by BP variation, independent of intrinsic stiffness. Changes in heart rate greatly affect variation in pulse waveform amplification from aortic segments to peripheral arteries, increasing the brachial systolic BP [26]. Heart rate in the multivariate model may mask variation in PWVao due to diabetes and hypertension status. When heart rate was removed from the model (data not shown), diabetes (β = 0.122, *p* = 0.027) and hypertension status (β = 0.207, *p* < 0.001) became independently associated with PWVao. CAVI, unlike PWVao, was not related to heart rate nor to BP in multivariate regression, even when systolic BP was substituted with diastolic, mean or pulse pressures (data not shown), as found elsewhere [27]. The capture of standard major CVD risk factors by CAVI is similar to other reports [20, 24, 28, 29]. Contrary to other studies [28–30], none of the biochemical parameters predicted variation in CAVI values here. This might be the result of a robust model; all the independent variables were simultaneously forced into the regression. The model attempts to mimic the complex interaction among various cardiovascular risk factors *in vivo*.

CAVI and haPWV had independent positive association with age but negative with BMI in all participants. The effect of age on CAVI was stronger in healthy participants, similar to Japanese studies, where CAVI increased at a rate of 0.5units/10 years in both men and women [31]. We speculate that the U-shape relationship between most anthropometric measurements and vascular health [32] may explain the negative association of anthropometric indices with CAVI and haPWV in our findings. The relationship between CAVI and body composition defined as true lean body or fat mass have been reported in a population-based study. Nagayama et al. [28] reported that CAVI was associated with baseline anthropometric indices and decreased after reduction in body weight by caloric restriction. The association between obesity and arterial stiffening might be attributed to insulin resistance, reduction in nitric oxide synthesis and pro-inflammatory state associated with obesity [33, 34].

Blood vessel damage in T2D is also characterised by resistance vessel hypertrophy and endothelial leakage (as in ‘micro’-albuminuria) [35]. Hypertension without diabetes is characterised by ‘eutrophic’ remodelling of resistance vessels *without* hypertrophy, at least, until decompensation occurs, leading to increased endothelial dysfunction and peripheral vascular resistance [36]. These pathophysiological trajectories are interlinked in several ways; T2D and hypertension induce changes in arterial structure through similar yet independent pathways [37]. The mechanism by which T2D per se contributes to increased arterial stiffness and hypertension is not yet certain [36]. However, decreased nitric oxide activity, activation of the renin-angiotensin system, mitogen activated kinase pathways, advanced glycated end-product generation and increased oxidative stress all seem to contribute in T2D [17, 38]. CAVI has recently been shown to correlate with oxidized lipoprotein(a) in older [30], but not younger [24], patients, and is associated with changes in oxidative stress status in T2D patients with abnormal levels of LDL [39]. However, the physiological mechanisms underlying elevated arterial stiffness in T2D and hypertensive patients in these Ghanaian patients will need biopsy and post-mortem studies to clarify tissue pathology.

The limitations of this study were its cross-sectional nature, hospital-based cases under treatment and the relatively small number of community-based healthy controls (*n* = 28), might give enough power for analysis. Future studies with prospective designs should provide valuable information about the utility of various indices of arterial stiffness in African populations.

## Conclusion

This study has shown indices of arterial stiffness, particularly, CAVI and haPWV, are useful tools for screening and discrimination of cardiovascular risk status in Ghanaian patients with T2D and hypertension. These indices may offer more precise targets for treatment than (higher) BP alone, particularly in low resource sub-Saharan African setting.

## Additional files

Additional file 1: Table S1. Anthropometric and Biochemical Characteristics of Subjects (mean ± SD). **Table S2.** Univariate Association of CAVI and various cardiovascular risk factors. **Table S3.** Univariate Association of Aortic PWV and various cardiovascular risk factors. **Table S4.** Univariate Association of heart-ankle PWV and various cardiovascular risk factors. (DOC 94 kb)
Additional file 2: Figure S1. Association between Cardio-ankle Vascular Index and Aortic PWV. **Figure S2.** Cardio-ankle Vascular Index at various Brachial Systolic BP levels. (DOC 89 kb)
Additional file 3: Dataset for the study. (XLSX 110 kb)

## Abbreviations

AIx: Augmentation index
BP: Blood pressure
CAVI: Cardio-ankle vascular index
haPWV: Heart-ankle pulse wave velocity
HTN: Hypertension
PWVao: Aortic pulse wave velocity
T2D: Type 2 diabetes
