# Supplementary material for: Arterial stiffness in hypertensive and type 2 diabetes patients in Ghana: comparison of the cardio-ankle vascular index and central aortic techniques
Source: BMC Endocr Disord. 2016 Sep 29;16:53. doi: 10.1186/s12902-016-0135-5 (PMC5041289; doi:10.1186/s12902-016-0135-5)
Supplement: Additional file 1: Table S1. — Anthropometric and Biochemical Characteristics of Subjects (mean ± SD). Table S2. Univariate Association of CAVI and various cardiovascular risk factors. Table S3. Univariate Association of Aortic PWV and various cardiovascular risk factors. Table S4. Univariate Association of heart-ankle PWV and various cardiovascular risk factors. (DOC 94 kb) [file 12902_2016_135_MOESM1_ESM.doc]

**Supplementary Tables (web-only):**

Table S1. Anthropometric and Biochemical Characteristics of Subjects **(mean±SD)**

| Characteristics | Controls  (n=28) | T2D only (n=49) | Hypertensive only (n=50) | Hypertensive T2D (n=143) |
| --- | --- | --- | --- | --- |
| Body fat (%) | 32.5±14.7 | 31.1±10 | 39.4±11.2^*#^ | 35.6±11.1^*#^ |
| Visceral fat (%) | 10.6±3.9 | 10.9±4.7 | 11±2.9 | 12.1±4.2 |
| Waist girth (cm) | 92±22.3 | 94.1±9.9 | 98.3±11 | 101.4±11.5 |
| Alcohol (%) | 19.0 | 8.1 | 5.1 | 6.2 |
| FPG (mmol/L) | 4.9±1.2 | 8.829±3.4*^§^ | 4.2±1.2 | 8.9±3.2*^§^ |
| 2h-PPG (mmol/L) | 6.8±1.9 |  | 6.9±2.3 |  |
| TC (mmol/L) | 5.6±1.2 | 4.3±1.4*^§^ | 5.2±1.422 | 4.4±1.3*^§^ |
| TG (mmol/L) | 1.2±0.5 | 1.1±0.6 | 1.2±0.7 | 1±0.5 |
| HDL (mmol/L) | 0.7±0.2 | 0.8±0.6 | 0.8±0.2 | 0.7±0.2 |
| LDL (mmol/L) | 3.8±1.3 | 2.8±1.3 | 3.4±1.7 | 3±1.1^*#^ |
| WHR | 0.9±0.2 | 0.9±0.1 | 0.9±0.1 | 0.9±0.1 |

T2D, type 2 diabetes; FPG, fasting plasma glucose; 2h-PPG, 2-hour post glucose-load plasma glucose; TC, cholesterol; TG, triglycerides; LDL, low density lipoprotein cholesterol; HDL, high-density lipoprotein cholesterol WHR, waist to hip ratio.

*vs Controls, p<0.05

§ vs Hypertensive only, p<0.05

# vs T2D only, p<0.05

Table S2. Univariate Association of CAVI and various cardiovascular risk factors.

|  | Entire group | | Control group | | Patient groups combined | |
| --- | --- | --- | --- | --- | --- | --- |
|  | r | p | r | p | r | p |
| **Age** | **0.61** | **<0.01** | **0.73** | **<0.01** | **0.59** | **<0.01** |
| BMI | -0.13 | 0.03 | -0.36 | 0.07 | -0.12 | 0.06 |
| Body fat | -0.1 | 0.09 | -0.18 | 0.34 | -0.13 | 0.04 |
| Visceral Fat | 0.05 | 0.49 | 0.12 | 0.56 | 0.03 | 0.64 |
| WHR | 0.28 | <0.01 | 0.35 | 0.09 | 0.33 | <0.01 |
| SBP | 0.02 | 0.79 | 0.19 | 0.34 | -0.04 | 0.57 |
| DBP | 0.1 | 0.1 | 0.3 | 0.12 | 0.01 | 0.86 |
| **PP** | **0.3** | **<0.01** | **0.07** | **0.71** | **0.27** | **<0.01** |
| **MBP** | **0.19** | **0.01** | **0.28** | **0.19** | **0.11** | **<0.09** |
| Heart rate | 0.07 | 0.29 | 0.06 | 0.76 | -0.01 | 0.96 |
| FPG | 0.12 | 0.05 | -0.11 | 0.95 | 0.06 | 0.34 |
| TC | -0.03 | 0.66 | 0.28 | 0.2 | 0.01 | 0.98 |
| TG | -0.03 | 0.62 | 0.41 | 0.03 | -0.05 | 0.46 |
| HDL | 0.01 | 0.85 | 0.19 | 0.36 | 0.01 | 0.99 |
| LDL | -0.02 | 0.64 | 0.11 | 0.6 | 0.01 | 0.84 |

BMI, body mass index; CAVI, cardio-ankle vascular index; SBP, systolic blood pressure; DBP, diastolic blood pressure; PP, pulse pressure; MBP, mean blood pressure; HR, heart rate; FPG, fasting plasma glucose; 2h-PPG, 2-hour post glucose-load plasma glucose; TC, cholesterol; TG, triglycerides; LDL, low density lipoprotein cholesterol; HDL, high-density lipoprotein cholesterol.

Table S3. Univariate Association of Aortic PWV and various cardiovascular risk factors.

| Groups | Entire group | | Control group | | Patient groups combined | |
| --- | --- | --- | --- | --- | --- | --- |
|  | r | p | r | p | r | p |
| **Age** | **0.3** | **<0.001** | **0.52** | **0.008** | **0.25** | **<0.001** |
| **BMI** | **0.18** | **0.004** | -0.1 | 0.638 | **0.21** | **0.002** |
| **Body fat** | **0.19** | **0.002** | -0.01 | 0.957 | **0.2** | **0.003** |
| WHR | 0.12 | 0.072 | 0.29 | 0.182 | 0.07 | 0.319 |
| **SBP** | **0.42** | **<0.001** | 0.07 | 0.72 | **0.4** | **<0.001** |
| **DBP** | **0.39** | **<0.001** | 0.07 | 0.726 | **0.37** | **<0.001** |
| **PP** | **0.3** | **<0.001** | 0.01 | 0.95 | **0.27** | **<0.001** |
| **MBP** | **0.42** | **<0.001** | 0.08 | 0.711 | **0.41** | **<0.001** |
| **Heart rate** | **0.3** | **<0.001** | 0.33 | 0.104 | **0.25** | **<0.001** |
| FPG | 0.04 | 0.572 | -0.22 | 0.286 | -0.01 | 0.903 |
| TC | 0.02 | 0.706 | 0.02 | 0.936 | 0.05 | 0.472 |
| TG | 0.01 | 0.927 | 0.29 | 0.154 | 0.04 | 0.611 |
| HDL | 0.03 | 0.606 | 0.22 | 0.284 | 0.02 | 0.77 |
| LDL | -0.04 | 0.516 | -0.03 | 0.876 | 0.01 | 0.931 |

BMI, body mass index; CAVI, cardio-ankle vascular index; SBP, systolic blood pressure; DBP, diastolic blood pressure; PP, pulse pressure; MBP, mean blood pressure; HR, heart rate; FPG, fasting plasma glucose; 2h-PPG, 2-hour post glucose-load plasma glucose; TC, cholesterol; TG, triglycerides; LDL, low density lipoprotein cholesterol; HDL, high-density lipoprotein cholesterol.

Table S4. Univariate Association of heart-ankle PWV and various cardiovascular risk factors.

|  | Entire group | | Control group | | Patient groups combined | |
| --- | --- | --- | --- | --- | --- | --- |
|  | r | p | r | p | r | p |
| Age | **0.46** | **<0.001** | **0.597** | **0.001** | **0.435** | **<0.001** |
| BMI | -0.04 | 0.484 | -0.360 | 0.077 | -0.022 | 0.735 |
| Body fat | -0.02 | 0.763 | -0.121 | 0.549 | -0.050 | 0.446 |
| Visceral Fat | 0.03 | 0.671 | 0.033 | 0.875 | 0.008 | 0.903 |
| **WHR** | **0.222** | **<0.001** | **0.289** | **0.171** | **0.262** | **<0.001** |
| **SBP** | **0.593** | **<0.001** | **0.556** | **0.003** | **0.552** | **<0.001** |
| **DBP** | **0.445** | **<0.001** | **0.614** | **0.001** | **0.380** | **<0.001** |
| **PP** | **0.52** | **<0.001** | **0.381** | **0.05** | **0.496** | **<0.001** |
| **MBP** | **0.554** | **<0.001** | **0.630** | **<0.001** | **0.500** | **<0.001** |
| Heart rate | 0.142 | 0.022 | -0.005 | 0.982 | 0.064 | 0.330 |
| FPG | 0.102 | 0.101 | 0.045 | 0.825 | 0.027 | 0.682 |
| TC | 0.027 | 0.672 | 0.190 | 0.352 | 0.082 | 0.218 |
| TG | 0.016 | 0.8 | 0.198 | 0.332 | 0.028 | 0.668 |
| HDL | -0.01 | 0.874 | 0.251 | 0.217 | -0.030 | 0.652 |
| LDL | 0.014 | 0.82 | 0.102 | 0.612 | 0.076 | 0.244 |

BMI, body mass index; CAVI, cardio-ankle vascular index; SBP, systolic blood pressure; DBP, diastolic blood pressure; PP, pulse pressure; MBP, mean blood pressure; HR, heart rate; FPG, fasting plasma glucose; 2h-PPG, 2-hour post glucose-load plasma glucose; TC, cholesterol; TG, triglycerides; LDL, low density lipoprotein cholesterol; HDL, high-density lipoprotein cholesterol.
