# Supplementary material for: Arterial stiffness in hypertensive and type 2 diabetes patients in Ghana: comparison of the cardio-ankle vascular index and central aortic techniques
Source: BMC Endocr Disord. 2016 Sep 29;16:53. doi: 10.1186/s12902-016-0135-5 (PMC5041289; doi:10.1186/s12902-016-0135-5)
Supplement: Additional file 2: Figure S1. — Association between Cardio-ankle Vascular Index and Aortic PWV. Figure S2. Cardio-ankle Vascular Index at various Brachial Systolic BP levels. (DOC 89 kb) [file 12902_2016_135_MOESM2_ESM.doc]

**Supplementary Figures (online only)**


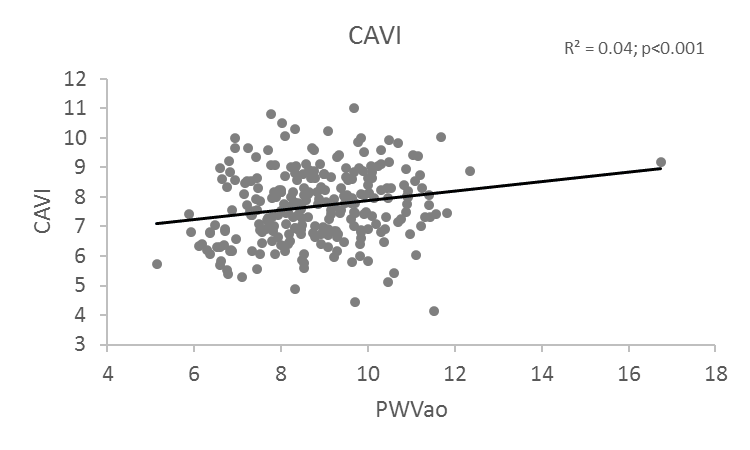


Figure S1. Association between Cardio-ankle Vascular Index and Aortic PWV.


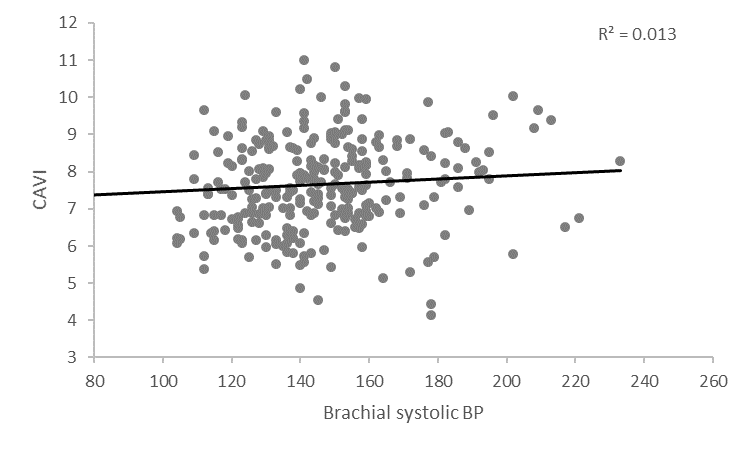


Figure S2. Cardio-ankle Vascular Index at various Brachial Systolic BP levels.
